# Multi-year Quantitative Evaluation of Stilbenoids Levels Among Selected Muscadine Grape Cultivars

**DOI:** 10.3390/molecules24050981

**Published:** 2019-03-11

**Authors:** Devaiah M. Kambiranda, Sheikh M. Basha, Stephen J. Stringer, James O. Obuya, Janana J. Snowden

**Affiliations:** 1 Southern University Agriculture Research and Extension Center, Ashford O. Williams Hall, Baton Rouge, LA 70813, USA; janana_snowden@subr.edu; 2 Center for Viticulture and Small Fruit Research, Florida A&M University, 6361 Mahan Drive, Tallahassee, FL 32308, USA; mehboob.sheikh@famu.edu (S.M.B.); jamesobuya@yahoo.com (J.O.O.); 3 USDA-ARS, Southern Horticultural Laboratory, 810 Hwy 26 West Poplarville, MS 39470, USA; stephen.stringer@ars.usda.gov

**Keywords:** muscadine grape, cultivars, HPLC, stilbenoids, variation

## Abstract

Stilbenoids such as *t*-piceid, *t*-resveratrol, ε-viniferins, and *t*-pterostilbene can differ significantly among grape cultivars and years due to variation in environmental conditions and subsequent stressors encountered during a year. This study evaluated diverse muscadine grape cultivars for their ability to consistently produce four major stilbenoids such as *t*-piceid, *t*-resveratrol, ε-viniferins, and *t*-pterostilbene irrespective of environmental changes that can impact their production. Berries from forty-two muscadine grape cultivars were collected for three years (2013, 2014, and 2015) to measure stilbenoids. Results showed significant differences in the composition of four stilbenoids among the muscadine cultivars. The highest level of stilbenoids was observed in ‘Fry Seedless’ (270.20 µg/g fresh weight) in each of the three consecutive years tested followed by ‘Pride’ (46.18 µg/g fresh weight) while ‘Doreen’ produced the lowest level of stilbenoids (1.73 µg/g fresh weight). Results demonstrated that certain muscadine grape cultivars consistently produced varied levels of the four major stilbenoids year after year. Based on the total content of stilbenoids, the 42 muscadine cultivars studied were grouped into three categories such as High, Medium and Low stilbenoid-containing cultivars. This information will help establish new vineyards with cultivars that are less prone to variations in environmental conditions and can consistently produce stilbenoid-rich muscadine grape berries with enhanced market value to promote consumer health.

## 1. Introduction

Plant-based foods are a rich source of phytochemicals *viz.* anthocyanins, glucosinolates, isoflavones, terpenoids, and phenolic compounds, etc. [1]. Some of the phytochemicals found in grapes are hydroxycinnamic acids, hydroxybenzoic acids, flavonoids, stilbenoids, and proanthocyanidins [2]. The muscadine grape (*Vitis rotundifolia*, Michx.) is native to the Southeastern U.S. and its berries and products can be utilized as inexpensive phytochemical-rich nutraceuticals or dietary supplements for prevention and treatment of chronic diseases in humans [3,4]. Numerous phytochemicals found in muscadine grapes are believed to provide numerous health benefits, including serving as anti-diabetic, anti-inflammatory, anti-cancer, anti-obesity, can reduce risks related to certain cancers, heart disease, and other age-associated disorders [5,6,7,8]. Among the polyphenol compounds found in skin, pulp, and seeds of muscadine grape berries, stilbenoids are of particular interest due to their health benefit properties [7,9,10,11,12,13]. Stilbenoids are formed via the phenylpropanoid and acetate-malonate pathway with stilbene synthase (StSy) catalyzing the formation of *trans*-resveratrol via condensation of 4-coumaroyl-CoA and malonyl-CoA. A dimethylated *trans*-pterostilbene [14], a 3-*O*-d-glucosidated *trans*- and *cis*-piceid [15] and peroxidation mediated oxidative dimerization and oligomerization end products, ε-viniferins [16], are some of the *t*-resveratrol derivatives identified. Several members of the family of stilbenoids including *t*-resveratrol (3,5,4-trihydroxystilbene), *t*-pterostilbene, and *t*-piceid are considered to confer nutraceutical benefits such as the treatment of cardiovascular disease and cancer [7,8,17]. Resveratrol (trans-3, 5, 4-trihydroxystilbene) was indicated to lower serum lipids [18], inhibit platelet aggregation [19], and can prevent the oxidation of low-density lipoprotein in humans [20]. 

Previous studies on stilbenoid content detected mostly *t*-resveratrol and *t*-piceid in muscadine grapes but did not include a multi-year study to determine the overall content for different muscadine cultivars [7,9,10,11,12,13]. Biosynthesis of stilbenoids in grapes occur via the phenylalanine pathway [16], in which the critical step is catalyzed by stilbene synthase [21,22]. The chemical structure of stilbenoids such as *t*-piceid, *t*-pterostilbene, ε-viniferins, etc. are built on the carbon-skeleton backbone of trans-resveratrol (3,5,4′-trihydroxystilbene) [23]. Thus, a high-performance liquid chromatography (HPLC) with ultraviolet (UV) has been used as a rapid analytical method for the quantification of stilbenoids [24].

To promote the muscadine grape as a stilbenoid-rich food crop, its major stilbenoids that include *t*-piceid, *t*-resveratrol, ε-viniferin, and *t*-pterostilbene must be stable. To overcome seasonal fluctuations, it is essential to evaluate existing muscadine cultivars to determine their ability to consistently express stilbenoids year after year under diverse environmental conditions. Thus, the study was conducted to quantify the major stilbenoids such as *t*-piceid, *t*-resveratrol, ε-viniferins, and *t*-pterostilbene in ripe berries that included 42 wine and table muscadine cultivars, harvested for three consecutive years to determine cultivars that can consistently produce a high content of stilbenoids.

## 2. Results

**Figure 1:**
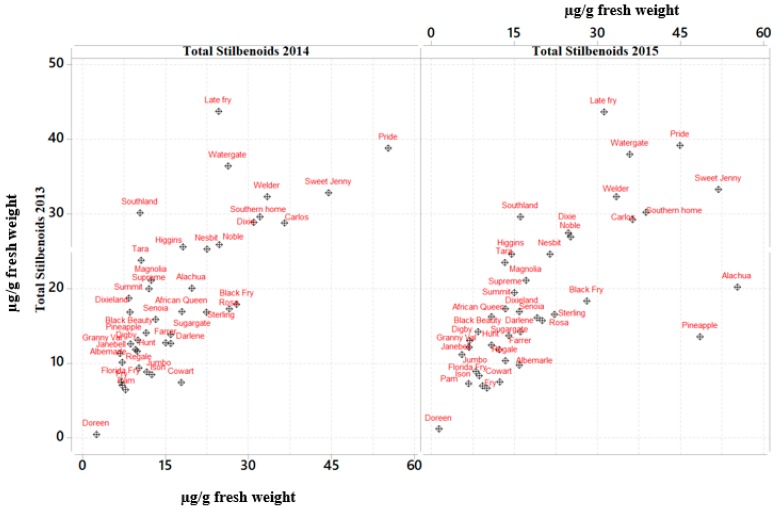
Scatter Plot showing differences in the total content of stilbenoids among muscadine cultivars.

Limited information exists on the extent of genetic variation and environmental impacts on the content of stilbenoids in muscadine grape cultivars. This study involved the evaluation of red and bronze wine and fresh fruit-type muscadine cultivars to determine the content of their stilbenoids for three consecutive years. Variations in content of stilbenoids were observed among the different cultivars and for each year (Figure 1). The total content of stilbenoids observed for three years showed a strong interaction for variety by year (Table 1). However, further analysis determined that bronze and red cultivars, such as Darlene, Welder, and Noble showed lower content of stilbenoids (Table S1 concentration of stilbenoids in wine and juice cultivars). Among the bronze table cultivars, Late Fry, Sweet Jenny, and Watergate showed higher berry stilbenoids’ content for all the three harvest seasons with a three-year average of 33.15, 42.68 and 33.16 µg/g of fresh weight respectively, while the lowest concentration of stilbenoids was determined from Florida Fry and Fry (7.22 and 8.13 µg/g fresh weight). Among red cultivars, African Queen (14.80 µg/g fresh weight), Southern Home (34.06 µg/g fresh weight) and Southland (19.13 µg/g fresh weight) followed by Sugargate and Supreme showed significantly higher levels of stilbenoids, whereas Ison and Jumbo contained the lowest level of stilbenoids for all the three years. 

### 2.1. Stilbenoid Composition 

Following HPLC analysis, four stilbenoids such as *t*-resveratrol, *t*-piceid, ε-viniferin, and *t*-pterostilbene were found in most muscadine cultivars (Table 2). Among the 42 cultivars studied, *t*-piceid and *t*-resveratrol were the major stilbenoids while ε-viniferin and *t*-pterostilbene were found in smaller) while the lowest amount was determined in ‘Doreen’ (1.73 µg/g fresh weight) (Table 2). 

### 2.2. t-Piceid 

**Figure 2:**
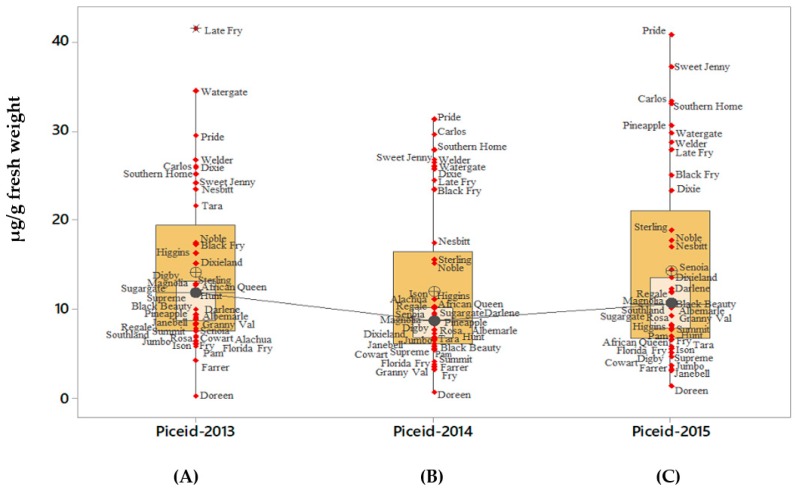
Variation of *t*-piceid content in different muscadine cultivars. The total *t*-piceid content was estimated for 41muscadine cultivars. The values presented in the boxplot format represents the data of 50% of the cultivars in the interquartile range, while the inside box represented 95% confidence interval for the median. The line connecting individual boxplot is the median line for the t-piceid content recorded for the three consecutive years 2013, 2014 and 2015. Each symbol represents the individual muscadine cultivar, the maximal, and the minimal values excluding outliers. Asterisks indicate the outliers. Symbols **A**, **B**, and **C** represent the average of total t-piceid obtained for the years 2013, 2014, and 2015 respectively by summing up the total t-piceid content for each variety obtained from three technical and biological replicates. Data obtained from cv. Fry Seedless have not been included in the figure.

*t*-Piceid was the predominant stilbenoids found among the 42 cultivars analyzed, and its berry concentration varied during the three years. For example, in a high stilbenoids’ content ‘Fry Seedless’, results determined variations in *t*-piceid concentration of 254; 209 and 233 µg/g fresh weight during the three consecutive years and with an average of 270 µg/g fresh weight (Table 1). Box plots showed considerable variation in *t*-piceid content as represented by the width of boxplot bars and the position of outliers (Figure 2). Results of *t*-piceid content showed a strong interaction for cultivars by year (Table 3). During the three years, some cultivars like ‘Late Fry’ and ‘Watergate’ produced high levels of *t*-piceid than the bulk of the population (Figure 2A, see dots on top of Boxplot for the year 2013). ‘Pride’ consistently produced the highest amount of *t*-piceid as compared to the rest of muscadine cultivars during the three years (Figure 2A–C, see dots on top of Boxplot for the years 2013–2015). ‘Doreen’ constantly produced the lowest amount of *t*-piceid during the three years (Figure 2A), see dots at bottom of Boxplot for the years 2013–2015).

### 2.3. t-Resveratrol 

**Figure 3:**
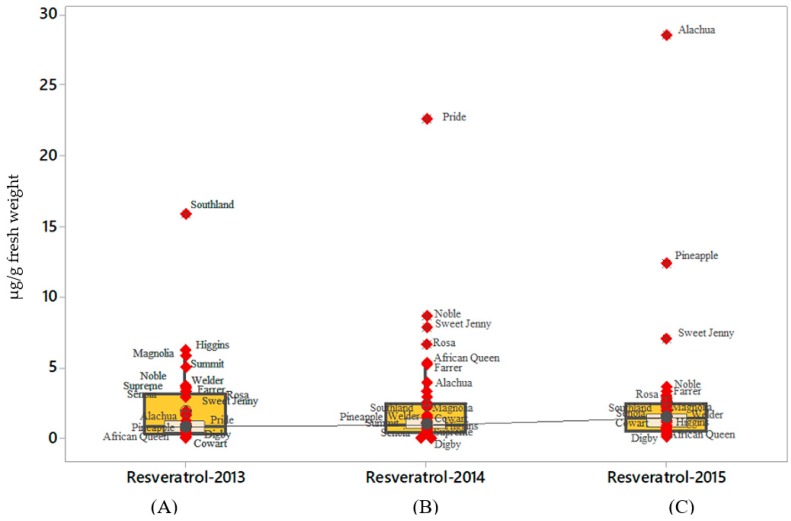
Total *t*-resveratrol content accumulated among different muscadine cultivars. The total *t*-resveratrol was analyzed for 41muscadine cultivars. The data presented in the boxplot indicated that *t*-resveratrol content for 50% of the cultivars was plotted in the interquartile range; whereas, the inside box represented 95% confidence interval for the median. The line connecting individual boxplot is the median line for the *t*-resveratrol content, which was estimated for the three consecutive years. Each symbol indicates individual muscadine variety and represents the maximal and minimal values excluding outliers. Asterisks indicate the outliers that are labeled in the figure. The boxplots **A**, **B**, and **C** in the figure showed the average total *t*-resveratrol content in the berries for 2013, 2014, and 2015 respectively. The data was obtained by summing up the *t*-resveratrol level for each muscadine variety and obtained using three technical and biological replicates. Data obtained from cv. Fry Seedless was not been included in the figure.

Unlike *t*-piceid, *t*-resveratrol concentration varied widely among the 42 cultivars analyzed as indicated by the outliers on each boxplot (Figure 3A–C). However, results indicated a lack of interaction for *t*-resveratrol content production by year (Table 4). *t*-Resveratrol content ranged between 15.84 µg/g fresh weight (‘Southland’) and 28.47 µg/g fresh weight (‘Alachua’) in berries harvested during 2013 and 2015 respectively. The lowest concentration (0.7 µg/g fresh weight) of *t*-resveratrol was found in ‘Cowart’ as indicated by the single outlier (Figure 3A), while cultivars such as ‘Albemarle’ and ‘Digby’ produced no *t*-resveratrol (Figure 3B) during 2014. ‘African Queen’ generated the lowest *t*-resveratrol of 0.67 µg/g fresh weight (Figure 3C) during 2015. The results demonstrated that *t*-resveratrol content in the berries varied during the three years and differences were attributed to grapevine exposure to varying environmental conditions and/or stressors.

### 2.4. Ɛ-Viniferin and t-Pterostilbene 

**Figure 4:**
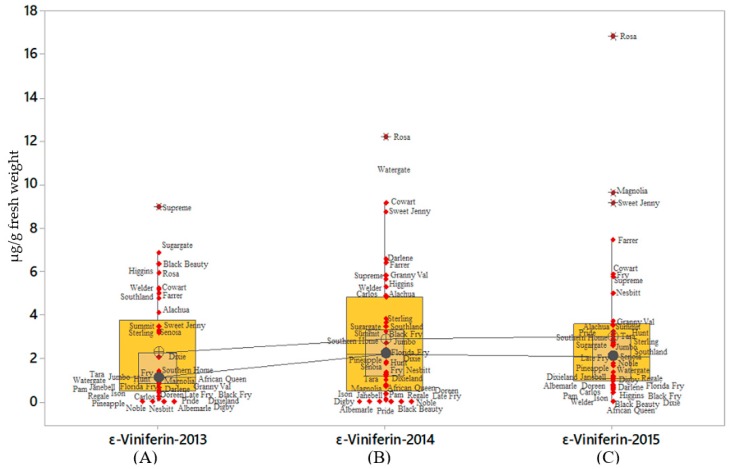
Levels of ε-viniferin present in various muscadine cultivars. The ε-viniferin accumulation in 41muscadine cultivars are presented in the boxplot format indicated that 50% of the cultivars plotted in the interquartile range; whereas, the inside box showed 95% confidence interval for the median. The line connecting individual boxplot is the median line for the ε-viniferin levels estimated for the three consecutive years. Each symbol indicates the individual variety and the maximal and the minimal content of ε-viniferin excluding outliers. Asterisks indicate the outliers. The boxplots **A**, **B**, and **C** in the figure showed the average of total ε-viniferin content in the berries for 2013, 2014, and 2015 respectively. The data was obtained by summing up the ε-viniferin content for each muscadine grape variety and obtained from three technical and biological replicates. Data obtained from cv. Fry Seedless have not been included in the figure.

**Figure 5:**
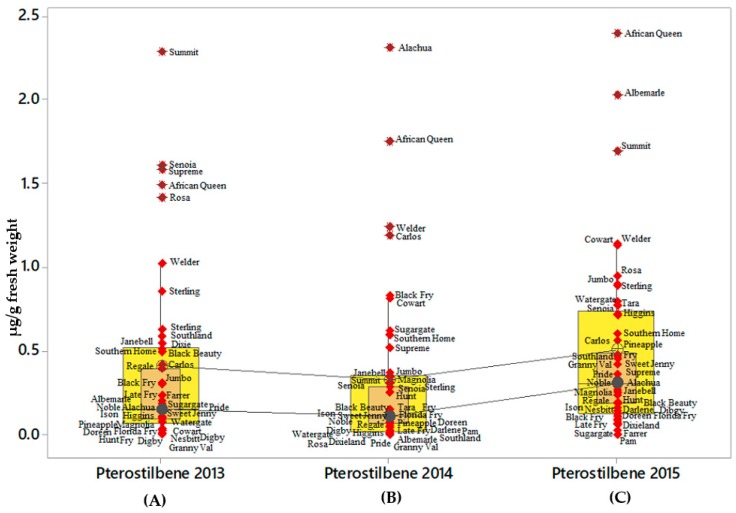
Differences in *t*-pterostilbene accumulation among muscadine cultivars. The analyzed *t*-pterostilbene levels from 41muscadine cultivars and plotted in the boxplots showed that 50% of the cultivars plotted in the interquartile range. The inside box showed a 95% confidence interval for the median. The line connecting individual boxplot is the median line for the *t*-pterostilbene levels estimated for the three consecutive years. The individual symbols show the variable data for each variety and the maximal and the minimal content levels excluding outliers are presented on the y-axis. Asterisks indicate the outliers. The boxplots **A**, **B**, and **C** in the figure are the average obtained from the total *t*-pterostilbene content in the berries for 2013, 2014, and 2015 respectively. The data was the sum of *t*-pterostilbene levels for each variety and obtained using three technical and biological replicates. Data obtained from cv. Fry Seedless have not been included in the figure.

Results of ε-viniferin content indicated a strong interaction for variety by year; however, pterostilbene content showed lack of interaction for muscadine grape cultivars by year (Table 5; Table 6). The distribution of ε-viniferin and *t*-pterostilbene was similar to that of *t*-resveratrol with highest ε-viniferin was found in ‘Supreme’ (2013), and ‘Rosa’ (2014 and 2015) with concentrations of 2.20, 12.20, and 6.49 µg/g fresh weight, respectively (Figure 4A–C). ‘Magnolia’ and ‘Sweet Jenny’ were part of the outlier ε-viniferin producers (Figure 4C). The distribution of concentration range for ε-viniferin was wide in 2014 when compared to the data observed for 2013 and 2015. The lowest ε-viniferin producers that formed lowest outlier on boxplot for all the three years were ‘Doreen’ (0.63 µg/g fresh weight) and ‘Digby’ (0.2 µg/g fresh weight) (Figure 4A–C). Generally, *t*-pterostilbene levels were low in all the muscadine grape cultivars. Despite the low levels of *t*-pterostilbene, a large number of cultivars were part of the outliers on the boxplots. The cv. ‘African Queen’ *t*-pterostilbene was an outlier in all the three harvest seasons followed by cv. ‘Summit’ in 2013 and 2015. Other cultivars like Alachua, Supreme, Albemarle, Senoia, Rosa, Welder, and Carlos were notably found among the outliers and were high *t*-pterostilbene producers. The results showed wide varietal differences with comparatively distinct levels of *t*-pterostilbene were among the outliers. (Figure 5A–C). 

## 3. Discussion

Stilbenoid-rich foods are widely consumed because of their perceived health benefits [4,10,17]. A wide range of plants across different genera and families have been reported to produce stilbenoids [25] under diverse conditions of growth and development [16,26,27,28]. It is important to note that stilbenoids are phytoalexins not constitutively expressed, which makes their content and composition uncertain in different plant tissues. The concentration of stilbenoids can vary as the berry forms, matures, and ripens. The study conducted by Gato et al. [29] determined that *t*-resveratrol levels increased from *veraison* to ripening phase. Furthermore, its content and composition are highly susceptible to environmental fluctuations, making the prediction of the total content of stilbenoids in different tissues somehow ambiguous. This was supported by our results that indicated that stilbenoids such as *t*-piceid, *t*-resveratrol, ε-viniferin, and *t*-pterostilbene showed strong interaction for muscadine grape cultivars by year (Table 1). Since stilbenoids are not readily soluble in aqueous media various solvents including methanol, ethyl acetate, acetone, and ethanol and extraction conditions such as total period needed to complete the process, temperature, and post-extraction incubation period [11,12,23] have been used with different rates of success to maximize the solubility of stilbenoids. Based on these reports, stilbenoids’ extraction protocol with methanol and ethyl acetate were used to recover the maximum yield of stilbenoids from muscadine grape and helped to avoid the ambiguity usually encountered while measuring its concentration. The extracted stilbenoids are expressed as µg/g of fresh weight for each berry, which represented the content of total stilbenoids in the whole muscadine berry. The analysis showed that concentration of stilbenoids from red and bronze muscadine cultivars showed a range between 1.73 and 246.53 µg/g fresh weight (Table. 2), which is consistent with previously reported data [11]. 

The study revealed that muscadine cultivars such as Fry Seedless, Hunt, Jumbo, Sugargate, Florida fry, Pam, Nesbit, Jumbo, Regale, and Noble produced similar amount of stilbenoids consistently during the three years; whereas, Alachua, Albemarle, Black beauty, Fry, Late Fry, Pride Supreme, Tara, and Watergate showed substantial variations in their stilbenoid content. The data suggest that some cultivars are less prone to environmental influences, and consistently maintained their stilbenoids’ levels (Supplementary Figure S3). It was determined that ‘Southland’, ‘Higgins’, ‘Magnolia’, ‘Supreme’, ‘Senoia’, and ‘Tara’ produced moderate levels of stilbenoids while ‘Digby’, ‘Ison’, ‘Fry’, ‘Janebell’, ‘Florida Fry’, ‘Pam’, and ‘Doreen’ produced the lowest concentrations. Interestingly, two of the nine high-stilbenoids containing cultivars such as Late Fry, and Sweet Jenny, [12] are preferred by consumers and, hence, may be of potential commercial value. Compositional analysis of whole berry stilbenoid extracts showed *t*-piceid as the major stilbenoids followed by *t*-resveratrol, ε-viniferin, and *t*-pterostilbene (Table. 1). Our finding is consistent with other reports that showed *t*-piceid is generally higher [30,31] in grapes than resveratrol [32]. Similarly, it was reported from a study conducted in Brazil whereby their results revealed that trans-piceid, trans-resveratrol, cis-resveratrol were present in high quantities (5 times more) than ε-viniferin [33]. 

The results were in conformity with the assumption that both genetic variations and environmental factors could impact the content of stilbenoids. The study conducted by Barchenger et al. [34] also reported that seasonal changes in temperature affected physiochemical content of muscadine grapes. Reduced content of stilbenoids can impact their potential value as nutraceuticals, an important attribute, which contributes toward health benefits and is mainly derived from the consumption of muscadine grapes and grape products.

## 4. Materials and Methods

### 4.1. Chemicals 

Stilbenoids standards *trans*-resveratrol, *trans*-piceid, *trans*-pterostilbene, *ε*-viniferin used for the identification of stilbenoids in grape berry extracts along with analytical grade methanol, acetonitrile, and ethyl acetate were purchased from Sigma-Aldrich (St. Louis, MO, USA). 

### 4.2. Berry Samples

Muscadine grape berries (42 cultivars) were sampled during vintage of 2013, 2014, and 2015 from the vineyard at Center for Viticulture and Small Fruit Research (located at 30.47 latitude and -84.17 longitude), Florida Agriculture and Mechanical University, Tallahassee, FL, USA. Ripe berries from EL-38 stage (modified E-L system, [35]) were collected from three different vines between August and September with recorded weather data (Table 7) during the harvest season. Berries collected from different clusters of the same vine from each cultivar represented one biological replicate. Three biological replicates from each cultivar were collected over three years.

The forty-two muscadine grape cultivars used in the study included: African Queen, Alachua, Albemarle, Black Beauty, Black Fry, Carlos, Cowart, Darlene, Digby, Dixie, Dixieland, Doreen, Farrer, Florida Fry, Fry, Fry Seedless, Granny Val, Higgins, Hunt, Ison, Janebell, Jumbo, Late Fry, Magnolia, Nesbit, Noble, Pam, Pineapple, Pride, Regale, Rosa, Senoia, Southern home, Southland, Sterling, Sugargate, Summit, Supreme, Sweet Jenny, Tara, Watergate, and Welder.

### 4.3. Extraction of Stilbenoids

The stilbenoids were extracted according to the described protocol [24]. Thirty berries collected from each vine, berries with seed were ground into a powder using liquid nitrogen. Ten grams (10 g) of ground sample was mixed with 100 mL of methanol and ethyl acetate (1:1, *v*/*v*) and homogenized for 10 min on a vortexer and further extracted for 24 h under darkness. The suspension was centrifuged at 10,000× *g* for 10 min. The supernatant was removed carefully, and the resulting residue was extracted a second time with 30 mL of methanol and ethyl acetate (1:1, *v*/*v*) as described above. The organic solvent extracts were pooled and vacuum-dried in a concentrator (SpeedVac concentrator, Savant Instruments, Inc. Holbrook, NY, USA) at room temperature in dark. The dried samples were then re-dissolved in 10 mL of HPLC grade methanol for quantitative measurement of stilbenoids using HPLC analysis.

### 4.4. Processing and Analysis of Stilbenoids

Each liquid extract was filtered through a 0.22 μm nylon membrane filter (Sartorius Stedim Biotech GmbH 37070 Gottingen Germany, Goettingen, Germany). High-performance liquid chromatography (HPLC) analysis of stilbenoids was carried out with an HPLC system equipped with a 2487 dual UV detector and 1525 gradient pump (Waters Corporation, Milford, MA, USA). The HPLC pumps, autosampler, and detectors were controlled via Waters Empower software (Empower 3 service pack 2) supplied by Waters Corporation (Milford, MA). The analytical column Luna RP C18 (4.6 × 250 mm; particle size, 5 um) and guard cartridge (C18 4 × 3.0 mm) were obtained from Phenomenex (Torrance, CA, USA). The column temperature was maintained at 25 °C. The gradient elution profile was as follows: 90% solvent water (B), 10% solvent acetonitrile (A) (0–18 min); 85% A, 15% B (18–23 min); 85% A 15% B (23–30 min); 10% A, 90% B (30–35 min). The flow rate was set at 0.4 mL/min. A volume of 2 µl of each sample was injected to resolve and measure individual stilbenoids. Three injections were performed in sequence for each biological replicate. UV absorbance detection was recorded using dual wavelengths at 285 and 305 nm. A mixture of the standards was prepared using 1, 2, 3, 4, 5, and 6 ng of each of the four stilbenoids and used for calibration and quantification of stilbenoids.

The standards, *t*-piceid, *t*-resveratrol, ε-viniferin, and *t*-pterostilbene (Sigma Aldrich, St. Louis, MO, USA) were prepared and used as described above. Samples and calibration standards were run in triplicates. Chromatograms were acquired and the area under the curves (AUC) was calculated using Empower III software (Waters Corporation, Milford, MA, USA). The linearity ranges of the calibration curves were R^2^ = 0.9906. Quantification of stilbenoids from the 42 muscadine cultivars was based on the calibration curves obtained from the respective standards. 

### 4.5. Representative Chromatogram of Stilbenoids’ Standards and Cultivars

The peak of the compounds eluted at different time points in the chromatogram represents *t*-piceid, *t*-resveratrol, ε-viniferin and *t*-pterostilbene (Supplementary Figures S2–S4). The total content of stilbenoids was obtained by the sum of the four isomers quantified using the standard curve method as described above.

### 4.6. Statistical Analysis

Statistical analyses were performed using Minitab software 17 (Minitab Inc, State College, PA, USA) and R statistical computing environment [36]. Differences in the total concentration of individual stilbenoids between cultivars were assessed using ANOVA Tukey post hoc test and comparison of means (*P* < 0.05) with GraphPad Prism 6.0 software (La Jolla, CA, USA). 

## 5. Conclusions

Knowing which specific muscadine grape cultivars that can consistently produce desirable levels of stilbenoids such as *t*-piceid, *t*-resveratrol, ε-viniferins, and *t*-pterostilbene is beneficial for value improvement and meeting the increasing demands of consumers and the health food industry. Additionally, this information would be useful toward the promotion a sustainable muscadine grape industry, including small farms, agro-pharmaceutical enterprises, and tailored breeding programs for the development of new cultivars with high phytochemical profiles to improve the growth of muscadine grape production in the southeastern US. 

## Figures and Tables

**Table 1:** Results of an analysis of variance (ANOVA) showing an interaction output for total content of stilbenoids by year from different muscadine grapes (2013–2015).

Source	DF	SS	Mean Sq.	F-Value	Pr (>F)
variety	40	11803	295.08	11.293	1.45 × 10^−12^
year	1	58	58.21	2.228	0.14319
cultivar:year	40	2250	56.25	2.153	0.00817
Residuals	41	1071	26.13		

**Table 2:** Total stilbenoids content of 42 muscadine cultivars for year (2013, 2014, and 2015). Concentration levels reported (µg/g fresh weight of the whole berry) are average values for the total stilbenoids content obtained from three biological replications.

Muscadine Cultivars	Brix°	2013 (µg/g Fresh Weight)	2014 (µg/g Fresh Weight)	2015 (µg/g Fresh Weight)	Mean (µg/g Fresh Weight)	SD
**African Queen**	17.5	15.95	17.6	10.87	14.81	3.51
**Alachua**	15.0	19.84	21	54.31	31.72	19.87
**Albemarle**	16.5	10.46	8.42	16.87	11.92	4.41
**Black Beauty**	19.0	13.3	10.95	9.23	11.16	2.04
**Black Fry**	17.0	18.61	28.83	26.86	24.77	5.42
**Carlos**	13.5	28.2	36.77	37.37	34.11	5.13
**Cowart**	17	7.91	16.73	11.32	11.99	4.45
**Darlene**	15.0	13.22	16.48	15.28	14.99	1.65
**Digby**	14.0	12.39	8.33	6.28	9	3.11
**Dixie**	14.5	28.41	29.8	24.59	27.6	2.70
**Dixieland**	14.5	15.94	8.45	15.6	13.33	4.23
**Doreen**	13.5	1.39	1.45	2.36	1.73	0.54
**Farrer**	15.0	11.97	14.81	13.05	13.28	1.43
**Florida Fry**	17.0	7.47	6.92	7.28	7.22	0.28
**Fry**	17.5	7.55	6.26	10.58	8.13	2.22
**Fry Seedless**	16.0	257.47	274.12	279.01	270.2	11.29
**Granny Val**	16.0	11.98	9.12	7.89	9.66	2.10
**Higgins**	16.5	25.48	17.94	13.19	18.87	6.20
**Hunt**	16.0	12.18	9.11	9.89	10.4	1.60
**Ison**	15.0	7.72	11.87	6.53	8.71	2.80
**Janebell**	13.0	10.45	7.81	4.21	7.49	3.13
**Jumbo**	14.5	8.38	10.65	8.83	9.29	1.20
**Late Fry**	17.5	43.7	25.32	30.43	33.15	9.49
**Magnolia**	13.0	21.9	11.28	15.75	16.31	5.33
**Nesbitt**	13.5	24.82	21.65	20.06	22.18	2.42
**Noble**	16.5	26.41	23.69	24.41	24.84	1.41
**Pam**	18.0	6.23	6.44	8.49	7.05	1.25
**Pineapple**	15.0	13.25	11.08	49.73	24.69	21.72
**Pride**	16.0	38.65	54	45.9	46.18	7.68
**Regale**	15.0	9.69	11	12.94	11.21	1.64
**Rosa**	13.0	16.3	26.1	19.67	20.69	4.98
**Senoia**	14.0	15.24	12.05	18.99	15.43	3.47
**Southern home**	16.0	29.48	33.17	39.54	34.07	5.09
**Southland**	17.0	30.3	11.12	15.97	19.13	9.97
**Sterling**	14.5	16.9	22.36	22.72	20.66	3.26
**Sugargate**	17.0	13.88	14.9	14.48	14.42	0.51
**Summit**	18.0	18.28	7.8	13.6	13.23	5.25
**Supreme**	17.0	19.33	13.05	14.88	15.75	3.23
**Sweet Jenny**	18.0	33.03	43.25	51.77	42.68	9.38
**Tara**	15.0	23.79	9.43	12.12	15.11	7.63
**Watergate**	14.5	37.17	27.31	35.02	33.17	5.18
**Welder**	14.0	33.12	32.9	33.19	33.07	0.15

**Table 3:** Results from an analysis of variance (ANOVA) showing an interaction output for *t*-piceid content by year from the different muscadine grape cultivars (2013–2015).

Source	DF	SS	Mean Sq.	F-Value	Pr(>F)
cultivar	40	9630	240.74	26.356	<2 × 10^−16^
year	1	1	1.23	0.134	0.71589
cultivar:year	40	898	22.46	2.459	0.00251
Residuals	41	375	9.13		

**Table 4:** Results of an analysis of variance (ANOVA) showing an interaction output for *t*-resveratrol content by year from the different muscadine grape cultivars (2013–2015).

Source	DF	SS	Mean Sq.	F-Value	Pr (>F)
variety	40	642.8	16.07	1.216	0.268
year	1	7.9	7.936	0.601	0.443
cultivar:year	40	595.5	14.887	1.127	0.352
Residuals	41	541.7	13.212		

**Table 5:** Results of an analysis of variance (ANOVA) showing an interaction output for ε-viniferin content by year from different muscadine grape cultivars (2013–2015).

Source	DF	SS	Mean Sq.	F-Value	Pr (>F)
cultivar	40	664.3	16.607	6.506	1.2 × 10^−8^
year	1	10.7	10.75	4.211	0.0466
cultivar:year	40	208.9	2.046	2.459	0.0124
Residuals	41	375	9.13		

**Table 6:** Results of an analysis of variance (ANOVA) showing an interaction output for *t*-pterostilbene content by year from different muscadine grape cultivars (2013–2015).

Source	DF	SS	Mean Sq.	F-Value	Pr (>F)
variety	40	19.442	0.4861	2.145	0.00844
year	1	0.184	0.1836	0.81	0.37337
cultivar:year	40	5.31	0.1328	0.586	0.95337
Residuals	41	9.293	0.2266		

**Table 7:** Recorded weather data in August and September (2013–2015) when berry samples were collected.

	Aug. 2013	Sept. 2013	Aug. 2014	Sept. 2014	Aug. 2015	Sept. 2015
**Avg. high temp.**	34.4 °C	31.7 °C	35.4 °C	31.3 °C	33.0 °C	32.2 °C
**Avg. low temp.**	23.7 °C	21.7 °C	23.1 °C	21.7 °C	23.1 °C	21.5 °C
**Precipitation**	15.42 cm	7.77 cm	5.74 cm	17.32 cm	21.89 cm	8.09 cm

## Supplementary Materials

Supplementary materials are available online. Figure S1. Chromatogram registered at 306 nm for stilbenoids standards; Figure S2. HPLC chromatogram registered at 306 nm in whole berry extracts for Pride cultivar; Figure S3. HPLC chromatogram registered at 306 nm in whole berry extracts for Pam cultivar; and Figure S4. Variation in stilbenoids’ content among the selected muscadine cultivars. Table S1. Concentration of stilbenoids produced in wine and juice type muscadines and Table S2. Concentration of stilbenoids produced in table type muscadines cultivars.
