# Supplementary material for: Multi-year Quantitative Evaluation of Stilbenoids Levels Among Selected Muscadine Grape Cultivars
Source: Molecules. 2019 Mar 11;24(5):981. doi: 10.3390/molecules24050981 (PMC6429103; doi:10.3390/molecules24050981)
Supplement: Supplementary file 1 [file molecules-24-00981-s001.pdf]

Supplementary Figures and Tables

Supplementary Figures

Figure S1.

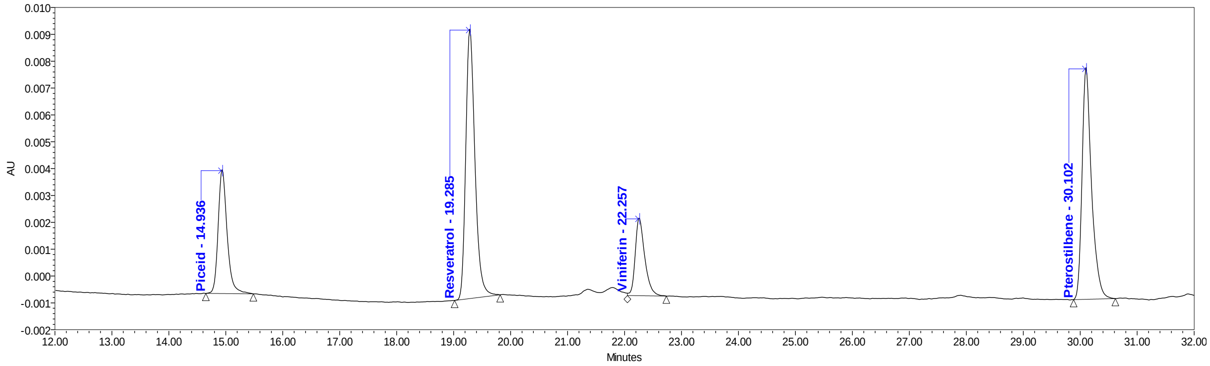

Chromatogram registered at 306 nm for stilbenoids standards

Figure S2

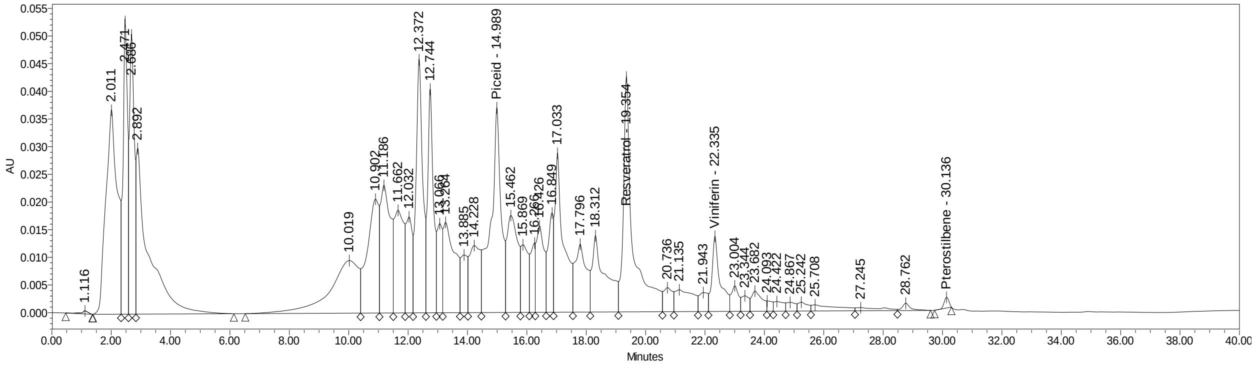

Chromatogram registered at 306 nm in whole berry extracts for Pride cultivar

Figure S3

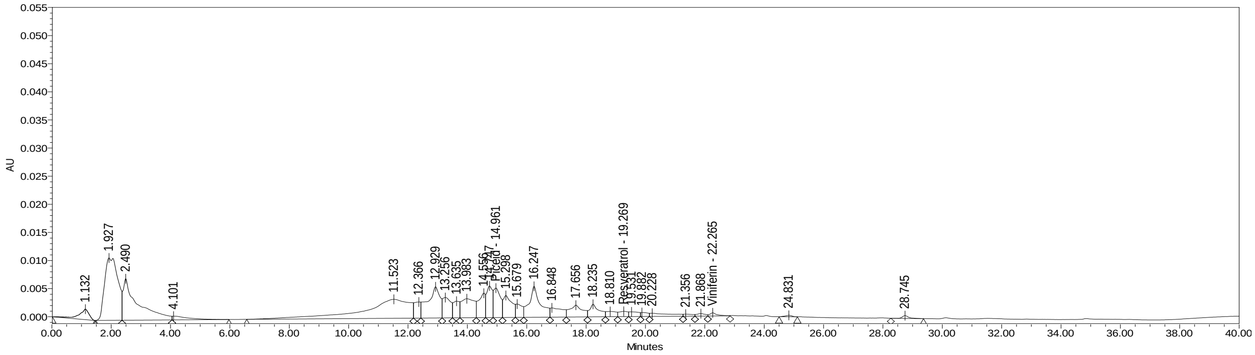

Chromatogram registered at 306 nm in whole berry extracts for Pam cultivar.

Figure S4

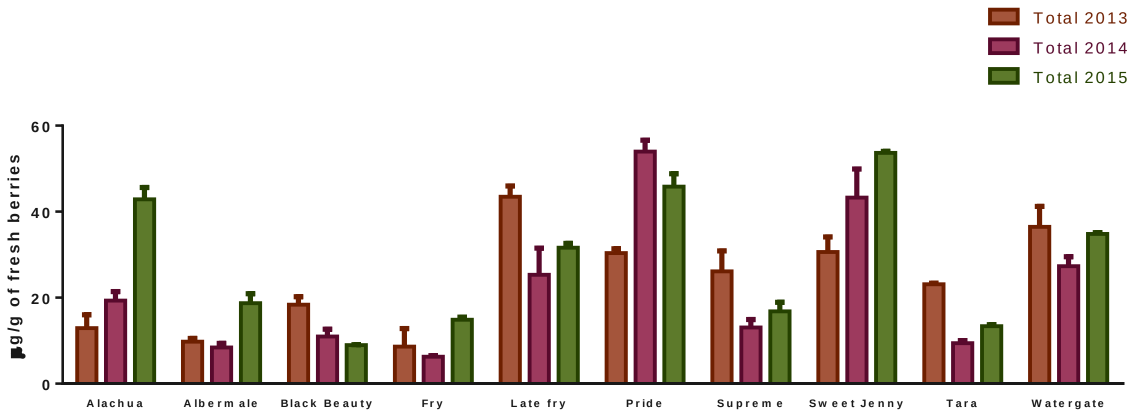

Variation in Stilbene content among the selected muscadine genotypes.

Supplementary Tables

Table 1. Concentration of stilbenes (µg/g fresh weight) produced in wine and juice type muscadines varieties.

MUSCADINE VARIETIES	TOTAL 2013	TOTAL 2014	TOTAL 2015
**BRONZE VARIETIES**			
**CARLOS**	28.20	36.77	37.37
**DOREEN**	1.39	1.45	2.36
**MAGNOLIA**	21.90	11.28	15.75
**STERLING**	16.90	22.36	22.72
**WELDER**	33.12	32.90	33.19
**PURPLE VARIETIES**			
**ALACHUA**	19.84	21.00	54.31
**COWART**	7.91	16.73	11.32
**NOBLE**	26.41	23.69	24.41
**REGALE**	9.69	11.00	12.94

Table. S2. Concentration of stilbenes (µg/g fresh weight) produced in table type muscadines varieties.

Muscadine Varieties	Total 2013	Total 2014	Total 2015
**Carlos**	28.20	36.77	37.37
**Doreen**	1.39	1.45	2.36
**Magnolia**	21.90	11.28	15.75
**Sterling**	16.90	22.36	22.72
**Welder**	33.12	32.90	33.19
**Alachua**	19.84	21.00	54.31
**Cowart**	7.91	16.73	11.32
**Noble**	26.41	23.69	24.41
**Regale**	9.69	11.00	12.94
